# Dental Quackery

**Published:** 1859-10

**Authors:** E. T. Wilson


### DENTAL QUACKERY.

### A PAPER READ BEFORE THE AMERICAN DENTAL CONVENTION.

BY E. T. WILSON, M. D. D. D. S.

THE prevalence of quackery in all departments of medical science, its rapid growth under the new systems evolved during the present century, and its novel and alarming ramifications in that branch of practice whose professors we are, affords a theme quite extensive enough for the present paper, and one that yields, even to the most ordinary observer, too many illustrations to present any difficulty except that of selection.

It cannot be denied that it is the duty of all regular practitioners, however onerous, unpleasant, or, in a pecuniary sense, unprofitable, that duty may appear to be, to wage incessant war against *irregularism*, or *quackery.* Every honorable man, in whatever station of life, should leave his testimony against dishonesty, and this is, in a peculiar sense, the duty of those to whom society entrusts its health and happiness.

It is a portion, and no small portion, of the pledge tacitly made by the youthful physician, surgeon or dentist, when he receives at the hands of the faculty a diploma of skill and learning, " that he will discountenance, and as far as in him lies, put down all who would pursue his road without the necessary qualifications. " Admitted that it may embroil him in unpleasant altercations, strifes and bickerings ;—admitted that the masses may not always distinguish between the philosopher, battling for a principle, and the empiric battling for a fee ;—the great interests of the science entrusted to his keeping, the dignity of wisdom and the future progress towards perfection of the profession he has chosen, demand of him that he will make no compromise with false professors, nor cease " to cry aloud and spare not," while unworthy Samaritans are within the temple gates. Under this impression I offer a few thoughts upon the progress of Dental Quackery, or Irregularism, with some suggestion as to the readiest method of opposing it. To the modern method of procuring business by means of advertising—a method so diametrically opposed to that of former times—much, very much of the quackery of the present day must be attributed. In the times which some of us remember, it was thought equally derogatory to the dentist as to the physician to advertise his profession. No long list of specified prices, from the " pulling of a tooth" to the " extracting of a fang," from " inserting a simple plug" to " the more difficult cases of the art," was considered professional. No curiously decorated signs—no bleached dental organisms, grinning horribly in glass cases, and exposed to the view of passers by—no unfraternal depreciation of fees, formed a portion of the Dentist's skill or duties. The Surgeon Dentist was supposed to be skillful and learned, and needed none of these appliances; he was supposed to be a gentleman and above their use. When his preparations for the practice of his art were made, he sat down in the locality he had chosen, and then, amidst his books and instruments, awaited in dignified silence that custom which, however slow, is always certain to the worthy professor. But now how different! The dental practitioner must study as well the structure of human brains as of human teeth. He must apply himself to the knowledge of mankind. It is allotted to him to find the soft and pulpy places of men's heads as well as their jaws. He must manufacture gas as well as teeth. The wax must be applied no less to idiosyncracies than to gums. If he is the fortunate possessor of a purse containing anything of value, it must be largely bled to pay the job printer, the sign painter, the card engraver. His time, so necessary to the study of his profession, must go in a generous proportion to the conventionalities of society; that, by art of many smirks and bows and hand-grips, he may win some customers. He is, in fact, a sort of curb-stone dentist. His mode of operating is superior to that of others. His pockets are well filled with evidences of his superior skill in the shape of sundry teeth, exhibiting a well filled crown or fang, and, possibly, an entire artificial denture may appear, to show his new invention and adaptation, which may be to him and to the heirs of his loins after him a dental immortality. Thus shall he live admired and die regretted.

Seriously, this is what the modern system of advertising has done for the dental profession, and I may say, for all other professions which have a scientific basis,—it has given to *publicity* that which is due to merit.

Before leaving this part of the subject I cannot forbear calling your attention to the wonderful rapidity with which men's characters increase under the influence of this system. Persons whose knowledge of teeth was confined, a few years since, to a few badly cleansed pegs in their own mouths, are enabled to wax great, like Jonah's gourd, in that little period, and count their patients by thousands. Formerly the growth, as well of the quack as the man of science, was a slow affair,—he must needs prove himself a man,—now he needs only prove himself an ingenious and a generous advertiser. Is it strange, then, that the patient and modest practitioner, whose share of public patronage has been earned by real merit, whose conscience has guided each instrument, and followed each filling,—is it strange that he should eye with disfavor those who have secured popularity by such unprofessional means.

If I were called upon to give the characteristics of an irregular dentist, in contradistinction to the opposite class, I would note two points of distinction. First, a disposition to follow the dictates of popularity rather than conscience; second, a disposition to win the way by means other than those of experience and merit. The evidences of the former are seen in a willingness to humor the whims of patients, to perform operations of temporary value only, to seize upon every novelty of the day at the expense of sound work, and generally to make the street and the forum a part of the operating room. Teeth and their connections form a most complicated organism, of which but little is known by the masses. With many persons "the application of cold steel is the sovereign cure" for all the ails pertaining to the teeth, and in that one popular notion consists the whole art of dentistry. Few imagine the intricacies with which the science abounds, and still fewer will make any allowance for the perplexities with which the wisest of the profession finds himself surrounded in the management of many a painful and complicated case. The *professional* dentist who has the courage to tell his patient that the resources of his art are exhausted and that he must dismiss him, is a hero of a sort, alas! too rarely found. The Dentist who will boldly refuse to extract a tooth, whose throbbings are filling day and night with pain, is a hero, of a humbler pattern, perhaps, than Alexander, but nevertheless a hero. These observations lead us to this conclusion, that in the popular ignorance upon the subject of dentistry, it is no wonder that quackery thrives. It is no wonder that every city, town and village swarms with "Surgeon Dentists," so called, of the character such as I have described. There are various grades of empirics, and I should be pleased, for one, if we could sound in their ears the true definition of "quack," for of all professions I am inclined to believe that ours is as much in advance of others as the dog was when he went out to pursue a bear. "Quack," I believe, comes from a Teutonic word "quacken," and to "quack" means "to cry like a duck," that is, to make a great deal of noise; to brag loudly and to talk ostentatiously.

But we have this feathered Bobadil at an advantage. He cannot advertise in the newspapers; he cannot condense his professional acquirements within an almanac; and, as his friends and fellow-citizens have no teeth to extract, he cannot exhibit his extraordinary talents by setting up as a dentist. So he is confined to his private puddle, where I am uncharitable enough to wish we could keep some of his featherless imitators, upon a low diet of their own amalgams. For, gentlemen, I am vain enough to believe that *our* quacks are more mischievous, more ignorant and more impudent than any others in the world. I will back almost any one of the noble army of self-styled "dentists," with his puffs about painless operations,—with his Golgotha of a show-case,—with his pocket full of stumps which he never extracted,—with his dentrifice, called by a hard Greek name, and which will probably extract teeth much faster than he can,—with his circulars, illustrated by magnified photographs of insects which are supposed to reside in and about the dental organism, until this scientific gentleman gives them notice to quit,—with his sliding scale of prices, always in accordance with the times,—I say I will back this miraculous person against quacks in the pulpit, quacks at the bar, in a word, quacks in general. I did intend to make an exception in favor of the scientific creatures who discover and dispense Vegetable Bitters, popularly supposed to be able to create an appetite under the ribs of death; but these gentlemen, it must be remembered, kindly alleviate the sufferings of those who have been deprived of the power of mastication, by taking away the desire or power to masticate at all: for if some of those unfortunate patients who have yielded to the seductions of puffs and powders and cheap artificial dentures, with a base of cheaper plate, were only as hungry as their mouths are hideous, I do not know which would be the most uncomfortable — their jaws or their stomachs. I think we may, without suspicion of maudlin sympathy, sincerely pity the sorrows of the person, whether old or young, who may have fallen into the hands of the individual whose acquirements I am sketching, or some more brilliant flourisher of the forceps who lives not by *skinning* his teeth, but by *skinning* the teeth of other people,—of some renovator, who gets his patients into a terrible scrape,—of some noisy blower of his own trumpet, who first fills the ears of his victims with words, and then *stuffs* their teeth with something a great deal worse, so that life for them can have no charms, except those which are purchased at the cost of perpetual and expensive etherization.

I do pity these unfortunates. I am not much given to the melting mood, except in my laboratory; but when I meet one of these victims, my sympathies are aroused in consequence of the delusion under which they have been laboring,—that they did not come to you or I, and then that he or she went to the other man, who undoubtedly told him that we were extortioners, charlatans, and clumsy fellows, because we do not furnish the community with almanacs; do not inform the world of our prodigious talents, through the medium of the newspapers; do not demand a certificate of skill every time we extract a tooth; do not keep a museum of our handiwork; nor do we make venerable teeth sound by the use of the "Odontalgic Rejuvenator." I suppose, gentlemen, upon the whole, it is much easier and much more profitable to be a quack.

I have known men who could scarcely tell the superior maxillary nerve from a chalk line, which they had been accustomed to use in their manipulations upon ship timber; neither could they tell the inferior dental nerve from a rope which should have encircled their necks. They could possibly distinguish between a *dental* and *copaiva* capsule, but did not know whether nature gives us thirty-two teeth or thirty-seven and a half; who could not distinguish between a cuspid and a molar tooth, and who supposed that the incisors were the small bones of the ear; who thought that great men have eight wisdom teeth,—men of ordinary talents five, and fools like themselves none at all. I have known such fellows to make money, dupes and fame, while gentlemen of worth, and scholarship and skill were barely making a living. The practitioner who makes a noise,—who has seven square feet of door-plate, two night-bells, one knocker, and a speaking tube,—who has certificates of his skill from presidents and parsons and governors and senators, who never saw him, and never want to see him,—who is mentioned in the newspapers as having on Thursday last performed a wonderful operation upon one of our first citizens,—amputating the *os hyoides*, excising the *parotid* gland, sawing out two-thirds of the lower jaw, and removing six ounces of tumor from the pharynx, after which the patient went home and ate a hearty dinner; that is the practitioner for the people's money. He scrapes, and pulls and excavates; he has his windows open that passers-by may smell ether, and listen to the howls of his victims; he becomes famous for giving the greatest amount of pain for the smallest amount of money; he works cheaply, talks cheaply, and is altogether a cheap person. When he is exposed, he cries out that he is persecuted; and he is all along sure that the time will come when people will be weary of exposing him. He goes on, however, puffing himself. He informs a world, suffering from toothache, that it can be cured in one-sixtieth of a second, and under no possible circumstances can it return. Price 12½ cents. Fangs extracted for 25 cents. Simple *soft* fillings inserted for two shillings; *amalgam* fillings, $1.00; gold filling, ditto. A small advance will be expected on all compound and internal fillings. He hangs an enormous and gilded tooth over his door, but forgets to add the well known inscription: "Leave hope behind you, you who enter here;" for if he had any caution to utter, it would be, "Don't forget to remember your pocket-books."

I dare say his victims are good people; but if their teeth are hard to extract, they are themselves susceptible of very soft influences; and I do not think it would do our friend any harm if he consulted his books, if he has any, before consulting the sign painter, and practice on the craniums of the departed, before he half killed the living. Something more is required in embracing our profession than a small stock of foil, an operating chair and a spittoon, half a pint of teeth, a lathe, a few ivory-handled pluggers, and the last new compound *screw* forcep, with a treatise on Dental Surgery on the side-table, and a bottle of ether on the shelf. I know that a first rate chair is very imposing, and that a case of shining instruments, which some take so much pride in displaying, has a thrilling effect upon the nerves of the patient. But I confess I never see a fine case of instruments, clean, bright, sharp and well arranged, without putting up a silent prayer that the same Providence which usually keeps little boys with their pockets full of matches from gunpowder, will interpose to preserve the whole collection from the hands of a blunderer.

You may have heard of the man who called himself a surgeon, because he owned a beautiful case of amputating instruments, and did not discover his error until he was prosecuted for cutting off the *wrong leg.* I do not care how fine a fiddle a man has,—its value does not enable him to rival Paganinni or Ole Bull.

The life and soul of a quack business is ostentation. A modest man will plant himself upon his merits, and will wait for the world to come to him; and I believe I am safe in saying that, as a general rule, the most meritorious man will be found to be the most modest. It is ignorance, and pretension, and assumption, and wild and headstrong arrogance which seeks by adventitious arts to atone for gross deficiencies; which attempts, by bluster and display, to blind society to professional incompetency. It is the want of dexterity and skill which impels a practitioner to tell what he can do, instead of doing it silently and surely, and trusting to his handiwork as the voucher for his ability.

A charlatan's horn may be very long, and its voice very loud, and his wind as endless as a New England north-easter, but he must have brains as well as lungs, if he would produce music; and perhaps he will do quite as well if he does not always make his own perfections the theme of his performances. But it is more sensible,—it will be more agreeable to our fellow-creatures, and we shall be more likely to make real advances in our profession, if we keep tolerably quiet, relying upon doing well whatever our hands may find to do. One patient, skillfully and permanently relieved, becomes your friend and patient forever after; and you will probably fill, extract and set for his whole family,—for his children, and if you live long enough, for his grand-children, to say nothing of the collateral advantage which you will derive from the smiles of his uncles, aunts and cousins. The best advertisement a dentist can have is a thorough piece of work, for his patient cannot smile or speak, cannot eat, and cannot even yawn without advertising him in the best, and puffing him in the only tolerable way. As for his patients, private and personal gratitude, there will be no end of it. When he eats his Thanksgiving dinner, when he speaks in the pulpit, the bar and the forum, or in any other public place, he sits down or rises only to call you blessed. The gratitude of such a friend is worth having, whether he pays his bill without growling, or does not pay it at all.

I am not sure that quackery has not flourished most formidably since the quacks ceased to be peripatetic and betook themselves to local habitations; I care not where; in Five Points or on the Fifth Avenue, in North street or under the shadow of the Massachusetts state-house. To be sure, an empiric is an empiric, whether he hides himself in a dingy den, with a private entrance for the modest, or puts out a shingle longer than himself in some more reputable locality. But a quack, with a local status, who catches one dupe, sometimes contrives to catch a great many before he finally explodes and vanishes and makes room for another ignoramus and blunderer, who will, in turn, make room for a third.

The dentist who attends militia musters and cattle shows; whose stock in trade consists of a bottle of colored acid and a bit of leather, and who cleans teeth in ten seconds, undoubtedly humbugs the bumpkins, but he does not cheat the best people, because they are not there. But put him, with his fluent tongue, his inexhaustible impudence and his sublime disregard of truth and morality, in some fashionable quarter of a great city, and he will be pretty sure to do irreparable injury to many teeth which a conscientious practitioner is interested in preserving.

It is all well enough to talk about intelligence, but I have known very intelligent people who took pecks of patent pills; swallowed gallons of panaceas, drenched themselves with oceans of infallible tinctures, wore magnetic rings, encased themselves in magnetic plasters, tortured themselves with galvanic batteries, and, finally, physicked themselves into another, and we hope a better land. I have known clergymen, (at least, men calling themselves such,) who certified to the almost incredible virtues of some concentrated bottle of fluid dirtiness,—who declared, over their own sign-manual, that they had been cured of dyspepsia, that one of their parishioners had been cured of a number of Job's comforters, another of typhoid fever, and a third of neuralgia, by the same precious compound.

These good men—I admit there are some honorable exceptions to the rule—have a tender regard for, and will puff almost any quack who has discovered a new dentifrice, a pretended painless method of operation, or some amalgam which put into the cavity of a tooth immediately turns to dentine; in return for which this grateful quack is ready to depopulate their own mouths and the mouths of their families, without the aggravation of a bill.

I suppose that I shall not get much credit for disinterestedness in declaring, but I do solemnly declare that they who thus play upon public credulity in one thing are not to be trusted in *anything*. I should be sorry to have any woman whom I loved or respected at the mercy of their coarse natures and their ether, chloroform or brandy. I would beseech the pure and good to shun them; to avoid them with a closer vigilance; since no wild outcry of anguish or shame can, after their infernal tampering, summon the good or the generous to the rescue of outraged honor. I am not speaking of what may be,—I am making plain allusions to what has been,—to facts. I do not know that such things should astonish us, for a man who will do *anything* for lucre will do *anything* for lust, if his base inclinations are in that lowest and most repulsive of all possible directions.

To conclude with a word or plan whereby unprofessional dentistry can be put down. This, you will admit, is a most difficult theme.

It has been said by a humorist of the last century that,

> "Oftimes the pleasure is as great
> Of being cheated as to cheat,"

and, while the world is constituted as it is, it were useless to expect that quackery, in any department of life, can be extirpated. Yet something may be done, both to check it and counteract its evils. I would suggest,—

First,—The highest possible standard of scientific attainment in our profession. To depreciate others we must appreciate ourselves. To exhibit quackery in its native hideousness to the world, we must remove ourselves as high as possible by professional acquirements from quackery, and make the space between us evident to the humblest mind. Were I at liberty to name them, I could designate from those around me, individual cases that might serve as patterns in all that is earnest, zealous and conscientious in dentistry.

Secondly,—A careful avoidance of any of the unprofessional arts to which I have alluded, and a recurrence to the primitive model of our fathers. Whosoever descends into the arena of popular competition will never rise to a dignified seat with the elders of our vocation.

Third,—Positive refusal to unite in consultation, or in any other manner associate with empirical practitioners.

I must remind you, as well as myself, of the great responsibilities which our profession entails. If dentistry be a distinct branch of medical science, the best medical men will agree with me in pronouncing it one not easily mastered; demanding great anatomical and physiological acquirements, intelligence, diagnostical talent, close observation, a clear head and a ready and steady hand; most successfully embraced by the honest, the thoughtful and the progressive; of vast importance to the general health of the body and to the enjoyment of all our physical faculties. If studious men have devoted their lives and their minds to diseases of the eye or of the ear; if many great medical minds have been solely employed in special fields of observation; surely, that which we have entered is wide enough for all our industry, for all our devotion, for all our strength. We have outlived, if I may so speak, the disreputable era of our profession. We have seen it acknowledged as important; we have proudly beheld our own land leading all others in this department of the curative art.

It is for us to maintain the respectability and the position which have been so hardly earned, by sternly discountenancing empiricism, whatever may be its form, however dazzling may be its assumptions, or however brilliant its temptations.

